# A Fuzzy-Based Fusion Method of Multimodal Sensor-Based Measurements for the Quantitative Evaluation of Eye Fatigue on 3D Displays

**DOI:** 10.3390/s150510825

**Published:** 2015-05-07

**Authors:** Jae Won Bang, Jong-Suk Choi, Hwan Heo, Kang Ryoung Park

**Affiliations:** Division of Electronics and Electrical Engineering, Dongguk University, 26 Pil-dong 3-ga, Jung-gu, Seoul 100-715, Korea; E-Mails: bangjw@dgu.edu (J.W.B.); jjongssuk@dgu.edu (J.-S.C.); gjghks@dgu.edu (H.H.)

**Keywords:** 3D human factor, variation of eye fatigue, fuzzy-based fusion method, 3D display

## Abstract

With the rapid increase of 3-dimensional (3D) content, considerable research related to the 3D human factor has been undertaken for quantitatively evaluating visual discomfort, including eye fatigue and dizziness, caused by viewing 3D content. Various modalities such as electroencephalograms (EEGs), biomedical signals, and eye responses have been investigated. However, the majority of the previous research has analyzed each modality separately to measure user eye fatigue. This cannot guarantee the credibility of the resulting eye fatigue evaluations. Therefore, we propose a new method for quantitatively evaluating eye fatigue related to 3D content by combining multimodal measurements. This research is novel for the following four reasons: first, for the evaluation of eye fatigue with high credibility on 3D displays, a fuzzy-based fusion method (FBFM) is proposed based on the multimodalities of EEG signals, eye blinking rate (BR), facial temperature (FT), and subjective evaluation (SE); second, to measure a more accurate variation of eye fatigue (before and after watching a 3D display), we obtain the quality scores of EEG signals, eye BR, FT and SE; third, for combining the values of the four modalities we obtain the optimal weights of the EEG signals BR, FT and SE using a fuzzy system based on quality scores; fourth, the quantitative level of the variation of eye fatigue is finally obtained using the weighted sum of the values measured by the four modalities. Experimental results confirm that the effectiveness of the proposed FBFM is greater than other conventional multimodal measurements. Moreover, the credibility of the variations of the eye fatigue using the FBFM before and after watching the 3D display is proven using a t-test and descriptive statistical analysis using effect size.

## 1. Introduction

With the rapid development of the 3-dimensional (3D) industry, significantly more people have access to 3D content from movies, games, home videos, and TV. Parallel to this development, the problem of eye fatigue with 3D displays has been identified, which is induced by the discrepancy between accommodation and convergence, binocular parallax, viewing distance, and viewing position [1]. Therefore, significant research for assessing eye fatigue has been initiated, which includes subjective evaluation-, camera-, and biosignal-based methods. This research on eye fatigue measurement can be classified into two categories: single modality-based and multiple modality-based methods.

The former methods measure eye fatigue using a single modality such as the image obtained with a camera [2,3] or using biosignals [4,5,6,7,8]. In previous studies, user eye blinks were measured using camera images for assessing eye fatigue [2,3]. Other studies proposed that visual fatigue could be measured using electroencephalogram (EEG) signals when watching 3D displays [4]. Chen *et al.* proved that the gravity frequency of the power spectrum and power spectral entropy of EEG signals can be used to measure visual fatigue for 2-dimensional (2D) TV and 3D TV [5]. Park *et al.* proposed that user electrocardiography (ECG) signals can be used for measuring visual fatigue while watching 3D TV [6]. In another study, EEG based on an event-related potential (ERP), was used to measure 3D visual fatigue [7]. Yu *et al.* proposed the method of measuring eye movement using electro-oculography (EOG) signals for evaluating visual fatigue on 2D and 3D displays [8]. However, none of the previous research based on single modality can guarantee the credibility of eye fatigue measurement because the performance of a single sensor can be influenced by various factors including face movement.

To measure eye fatigue with higher credibility, multiple modality-based methods based on multiple modality sensors have been proposed [9,10,11,12]. In a previous study, video-oculography (VOG) and EOG were used for measuring visual fatigue on 3D images [9]. Bang *et al.* proposed a method of measuring eye fatigue with 3D displays using EEG, eye blinking rate (BR), facial temperature (FT), and subjective evaluation (SE) [10]. In [11], ECG sensors, galvanic skin response (GSR), and skin temperature (SKT) with SE were used to measure eye fatigue on 2D and 3D displays. The power of the beta bands of EEGs and BRs, and a Bayesian network have been utilized for measuring eye fatigue when viewing 3D displays [12]. Although the multiple modality-based methods enhance the credibility of eye fatigue measurement compared to single modality-based methods, they have not combine the measured values from multiple modalities. Moreover, they have not considered the qualities of the measured values.

Therefore, we propose a new method for quantitatively evaluating the variation of eye fatigue before and after watching 3D displays by combining multimodal measurements. For the evaluation of the variation of eye fatigue with high credibility on 3D displays, a fuzzy-based fusion method (FBFM) is proposed based on the multimodalities of EEG signals, eye BR, FT, and SE. To measure a more accurate variation of eye fatigue, we obtain the quality scores of EEG signals, eye BR, FT, and SE. For combining the values of the four modalities, we obtain the optimal weights of the EEG signals, BR, FT, and SE using a fuzzy system based on the quality scores. Then, the quantitative level of the variation of eye fatigue is finally obtained using the weighted sum of the values measured by the four modalities. In Table 1, we show the comparisons of previous and proposed methods to measure eye fatigue.

**Table 1:** Comparison of previous and proposed methods to measure eye fatigue.

Category		Method	Advantages	Disadvantages
Single modality-based methods	Camera-based method [2,3]	Eye blink is measured	- Less influenced by movement of muscle, head, or body because of contactless method	- Data capturing speed is less than biosignal-based method
	Biosignal-based method [4,5,6,7,8]	EEG [4,5,7], ECG [6], and EOG [8] are measured	- Causes less discomfort to user than multiple modality-based methods because of smaller number of sensors attached to body	- Contains noise caused by movements of muscle, head, or body
Multiple modality-based methods	Method not combining information of multiple modalities [9,10,11,12]	VOG and EOG [9], EEG, BR, and FT [10], ECG, GSR, and SKT [11], and EEG and BR [12] are measured	- Measurement of eye fatigue is more accurate with multiple modalities than with single modality	- Does not consider qualities and weights of measured values
	Method combining information of multiple modalities **(proposed method)**	Combining multiple modalities based on FBFM considering quality of the measured values	- Accuracy of eye fatigue measurement is enhanced by combining multiple modalities considering the quality of the measured values	- Additional procedures are necessary for quality measures and combining multiple modalities

The remainder of this paper is organized as follows: in Section 2, the proposed system and the analysis of the quality measurements are described. In Section 3, the experimental setup and results are discussed. Finally, the conclusions are presented in Section 4.

## 2. Proposed System and Method

### 2.1. Proposed Method

**Figure 1:**
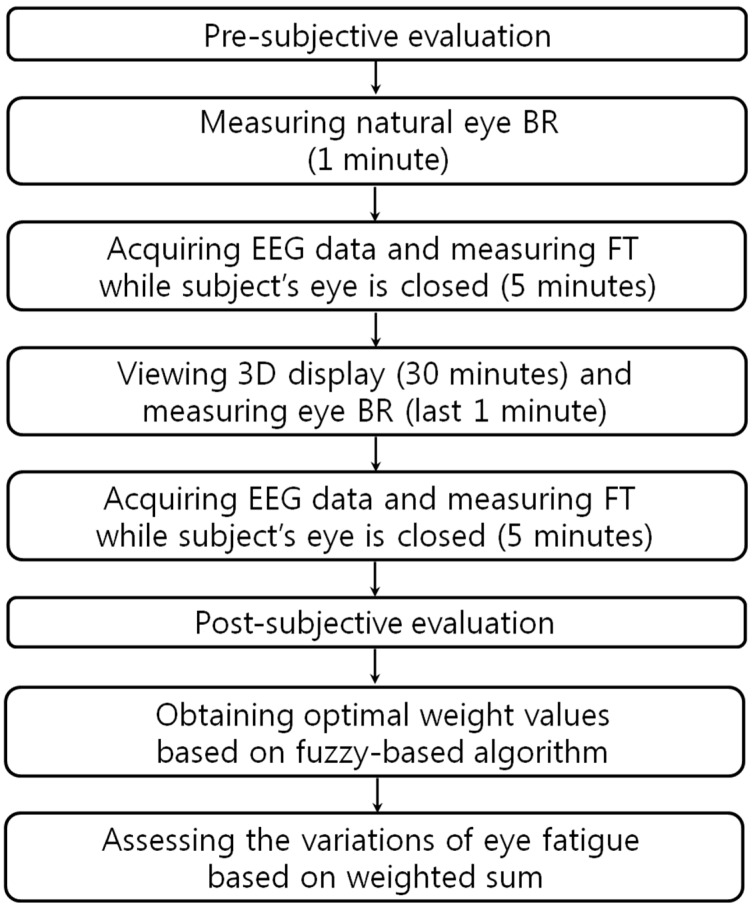
Flow chart of proposed method.

Figure 1 presents the proposed method for FBFM for multiple modalities considering the quality values of the modalities. To measure the SE before watching the 3D content, the subject’s condition is verified with a questionnaire regarding watching 3D content. To compare the eye BR before and after watching 3D content, the normal eye BR of the subjects is measured for one minute before watching the 3D content. Then, we proceed with the phase of measuring the subject’s EEG data and FT for five minutes with eyes closed to minimize any external visual stimuli that could influence the EEG data. Next, the subjects watch the 3D content for 30 min. The eye BR is measured for the final one minute of the 3D content watching period to obtain an accurate comparison of the variation of the eye BR before and after watching the 3D content. After watching 3D content, to compare the variations of the EEG data and FT before and after watching 3D content, the subject’s EEG data and FT are measured again for five minutes with eyes closed. Finally, the subject’s condition is again verified using an SE questionnaire.

With the measured values of the variations of SE, BR, EEG, and FT (before and after watching the 3D display), we obtain optimal weight values using a fuzzy algorithm employing the quality measurements for evaluating the confidence level of each modality. We assess the variations of eye fatigue based on the weighted sum of the calculated weight values based on the fuzzy algorithm and the acquired values of each modality.

**Figure 2:**
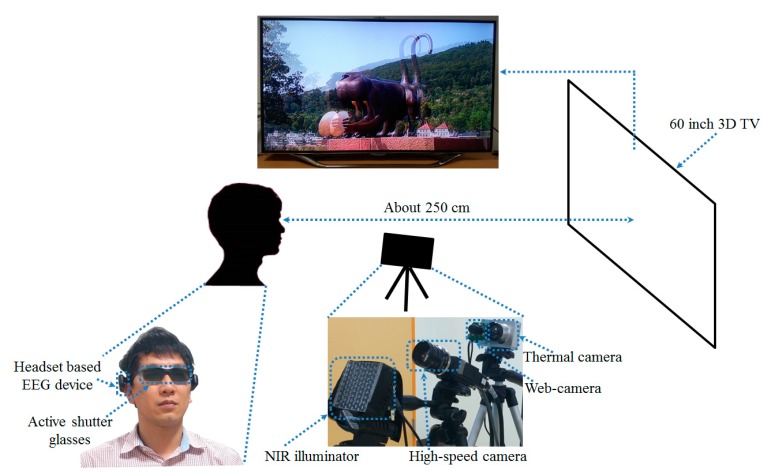
Proposed system for measuring eye fatigue on 3D display.

Figure 2 displays the proposed system for measuring the variations of eye fatigue before and after watching a 3D display. The detailed specifications and set-up of the proposed system can be found in [10]. As illustrated in Figure 2, the user wears a headset-based EEG measurement device and active shutter glasses [13] for watching the 3D content presented on a 60-inch smart TV [14] with the resolution of 1920 × 1080 pixels. To assess eye fatigue, we acquire EEG signals, eye BR, and FT using the EEG device, high-speed camera [15], and thermal camera [16], respectively. As indicated in Figure 2, the distance between the subject and the 3D TV is approximately 250 cm based on the safety distance guidelines for watching 3D TV [17]. The distance from the subject to the near-infrared (NIR) light illuminator is approximately 60 cm and to the high-speed camera about 80 cm. The distance between the subject and the thermal camera is about 100 cm.

### 2.2. Measurement Device and Analysis of EEG Data for Measuring Eye Fatigue

**Figure 3:**
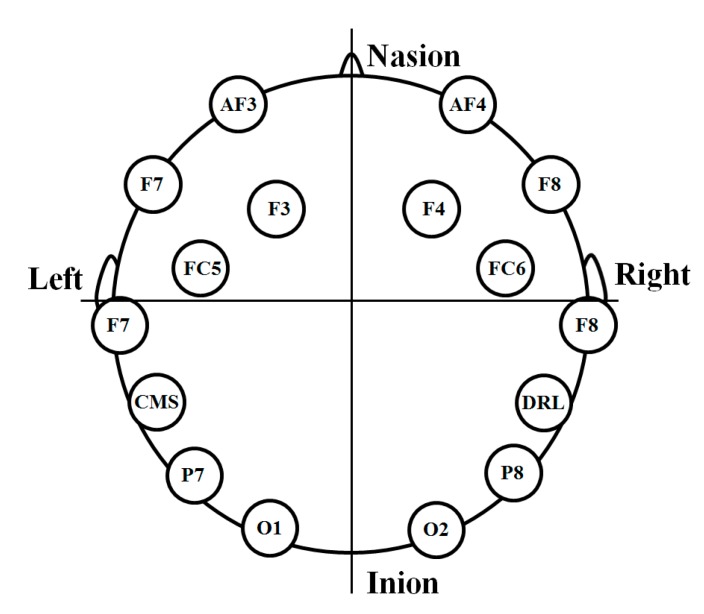
Location of scalp electrodes based on international 10–20 system.

The detailed specifications of the EEG measurement device and explanations of the EEG measurement methods in the proposed system can be found in [10]. To measure eye fatigue, the beta band (13–30 Hz) in the frequency domain of the EEG data is analyzed because watching 3D content is known to influence the power of the EEG signals in the beta band. The power of the EEG signals in the beta band is stronger while watching 3D content [6,7]. In the proposed system, a commercial device for measuring the EEG signals is used, and the name of this device is Emotive EPOC. The Emotiv EPOC headset has two reference nodes and 14 other electrodes [18]. The location of the scalp electrodes conforms with the international 10–20 system as indicated in Figure 3 [19,20]. EEG data processed using the built-in digital 5th-order Sinc filter is acquired at the sampling rate of 128 Hz (128 samples/s) [19,21].

The EEG signals are measured based on the micro-voltage levels from 14 nodes excluding two reference nodes (CMS and DRL). The DC levels of the measured EEG signals are adjusted for normalization and then the range of the EEG signals are rearranged from −1 to 1 using min-max scaling. The EEG signals are changed into those of a frequency domain using a Fourier transform with a window length of 128 samples [10]. In our previous research [10], the most dominant node for representing the variations of eye fatigue before and after watching a 3D display was experimentally selected as P7. Therefore, we use the variations of the EEG signals from the P7 node for measuring the variations of eye fatigue in our research.

### 2.3. Measurement Device and Analysis of BR for Measuring Eye Fatigue

Eye BR has been associated with eye fatigue. Eye BR is known to increase as a user feels elevated eye fatigue [22,23]. Detailed specifications of the camera device for the BR measurement in the proposed system can be found in [10]. To measure the eye BR of the subjects, a high-speed four megapixel (2048 × 2048 pixels) camera with capturing speed of 150 frames per second (fps) is used for acquiring images of both eyes [15]. The images are actually acquired at a lower speed (than 150 fps) because of the saving time of the images on the computer hard disk. Further, only a portion of the complete image (2048 × 512 pixels) is saved; only the region including both eyes is required for the BR measurement. In fact, the image sequences are obtained at a speed of 73.55 fps. Using a non-wearable camera system for BR measurement as illustrated in Figure 2, the user’s convenience is enhanced even when watching a display for a long time.

To measure the BR, an accurate detection of the pupil region is necessary. For the accurate detection of the pupil region, which is robust to the variations of environmental light, we use an NIR illuminator consisting of a high-power 8 × 8 NIR emitter with wavelength of 850 nm as indicated in Figure 2 [24]. Using an NIR illuminator of 850 nm does not dazzle the user’s eyes.

**Figure 4:**
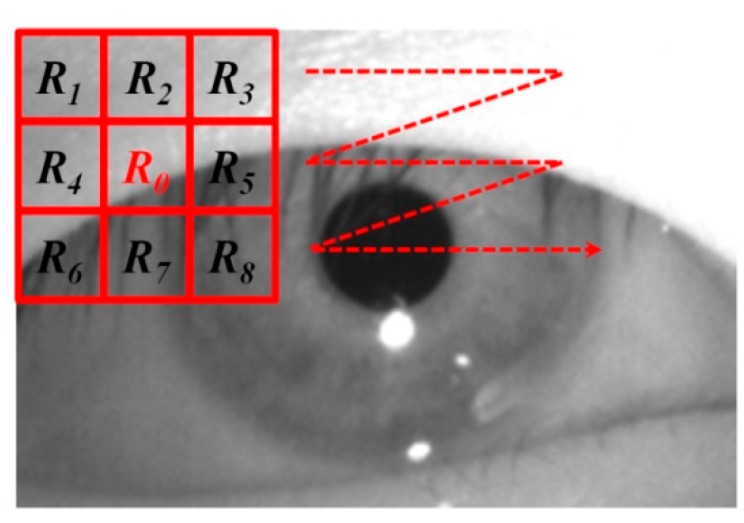
Procedure of sub-block-based template matching in the ROI.

The detailed methods of pupil detection and BR measurement can be found in [10]. To locate the pupil region, we detect the region of the corneal specular reflection (SR) (which is produced on the corneal surface by the NIR illuminator) using image binarization. Based on the detected region of the corneal SR, we set a region of interest (ROI) for pupil detection. To detect two areas of the pupil, sub-block-based template matching is used in the ROI [10,25]. The sub-block-based template matching algorithm uses a 3 × 3 mask including nine sub-blocks (*R_0_*~*R_8_*) as indicated in Figure 4. At each position of the mask in the ROI region, the mean of each sub-block (*R_0_*~*R_8_*) is calculated and the sum of the difference values is calculated between the mean of *R_0_* and those of *R_1_~R_8_*. This procedure is repeated by moving the mask, overlapping one pixel in both the horizontal and vertical direction.

**Figure 5:**
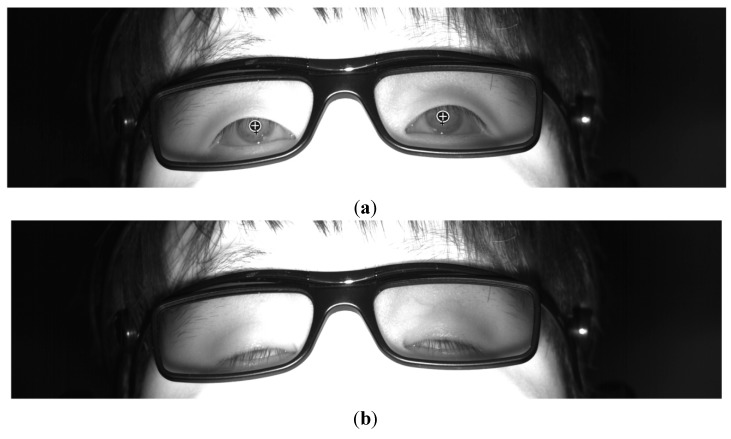
Result of determining eye openness and closure, (**a**) Open eyes; (**b**) Closed eyes.

If the *R_0_* region matches the pupil region, the sum of the difference values is maximized because the pupil region is usually darker than the surrounding regions as illustrated in Figure 4. Using the sub-block-based template matching method, a rough position of the pupil region is located. Then, the accurate boundary and center of the pupil are detected using image binarization and an ellipse-fitting method as depicted in Figure 5. If the boundary and center of the pupil are successfully detected by the ellipse-fitting method, the user’s eye is determined to be open; otherwise, it is determined to be closed. The eye BR is measured by counting the number of closed eyes for a time duration of one minute.

### 2.4. Measurement Device and Analysis of FT for Measuring Eye Fatigue

The detailed specification of the thermal camera and methods for measuring FT can be found in [10]. The infrared (IR) spectrum is classified into four sub-bands, NIR (wavelength ranges from 0.75 to 1.4 μm), short wave IR (SWIR, wavelength ranges from 1.4 to 3 μm), medium wave IR (MWIR, wavelength ranges from 3 to 8 μm), and long wave IR (LWIR, wavelength ranges from 8 to 15 μm) [26]. A considerable amount of heat energy is emitted from the MWIR and LWIR sub-bands. Therefore, these sub-bands are usually adopted for sensing human face temperature and are called the thermal sub-band [26]. In this research we call the image captured from this sub-band the thermal image.

**Figure 6:**
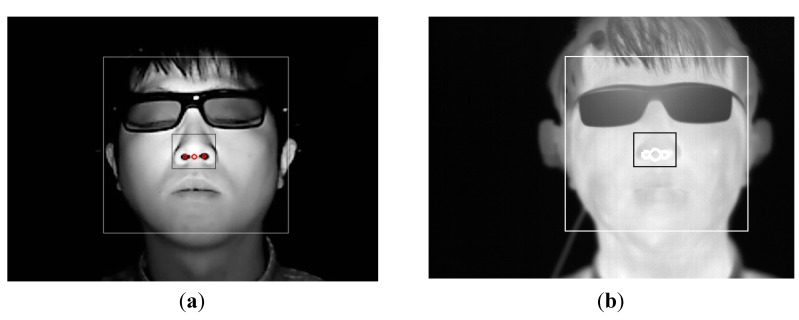
Detected regions of face and nose, (**a**) Detected regions of face and nose in the web-camera image; (**b**) Obtained regions of the face and nose in the thermal image using the geometric transform matrix based on the detected regions in web-camera image.

**Figure 7:**
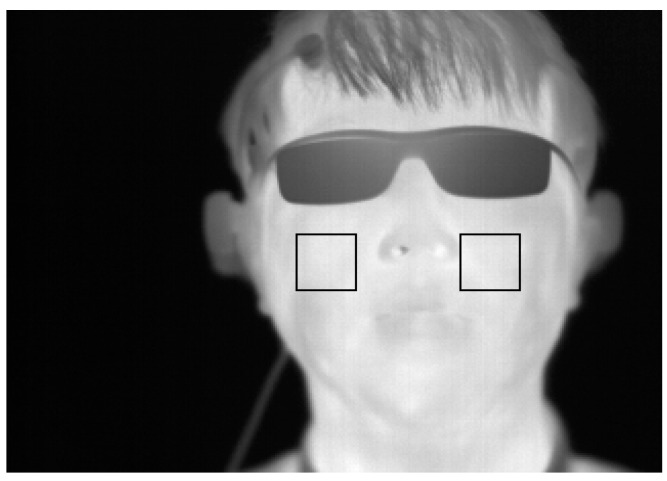
Cheek regions that are used for comparing the variation of FT before and after watching 3D content.

To measure the variation of FT before and after watching 3D content, a thermal camera with a resolution of 320 × 240 pixels of 14-bits is used, as illustrated in Figure 2. The temperature range of the thermal camera is −20 °C to 100 °C with an accuracy of ±1 °C or ±1% [16]. In a thermal image, it is difficult to detect an accurate position of the facial features because facial features are not distinctive in a thermal image, as indicated in Figure 6 and Figure 7. Therefore, a commercial web-camera (Webcam C600) [27] is attached next to the thermal camera as illustrated in Figure 2. The web-camera captures a 24-bit image of 800 × 600 pixels at a speed of 30 fps. The NIR illuminator is used to reduce the variations of the environmental illumination and stabilize the web-camera image.

The coordinates of the two images, web-camera and thermal camera, are set to be coincident based on the geometric transform depicted in Equation (1). A pair of four points ((*M_x0_*, *M_yo_*), (*M_x1_*, *M_y1_*), (*M_x2_*, *M_y2_*), (*M_x3_*, *M_y3_*) and (*N_x0_*, *N_yo_*), (*N_x1_*, *N_y1_*), (*N_x2_*, *N_y2_*), (*N_x3_*, *N_y3_*)) are necessary for calculating the eight unknown parameters (*a*, *b*, … *h*) in the matrix of the geometric transform. This pair of four ground-truth points is manually obtained from the captured images using a calibration board. The procedure of obtaining the matrix of the geometric transform is performed once, when the two cameras are combined. It is not necessary to repeat this procedure:

(1) $$\left[\begin{array}{c} \begin{array}{cc} N_{x0} & N_{x1}\\ N_{y0} & N_{y1} \end{array}\;\begin{array}{cc} \;\;N_{x2} & N_{x3}\\ \;\;N_{y2} & N_{y3} \end{array}\\ \begin{array}{cc} 0\; & 0\\ 0\; & 0 \end{array}\;\begin{array}{cc} \;0 & \;0\\ \;0 & \;0 \end{array} \end{array}\right]=\left[\begin{array}{c} \begin{array}{cc} a & b\\ e & f \end{array}\;\begin{array}{cc} \;c & d\\ \;g & h \end{array}\\ \begin{array}{cc} 0 & 0\\ 0 & 0 \end{array}\;\begin{array}{cc} \;0 & 0\\ \;0 & 0 \end{array} \end{array}\right]\left[\begin{array}{c} \begin{array}{cc} M_{x0}\; & M_{x1}\\ M_{y0}\; & M_{y1} \end{array}\;\begin{array}{cc} \;\;\;\;\;\;\;\;M_{x2}\; & M_{x3}\\ \;\;M_{y2}\; & M_{y3} \end{array}\\ \begin{array}{cc} M_{x0}M_{y0} & M_{x1}M_{y1}\\ 1 & 1 \end{array}\;\begin{array}{cc} \;\;M_{x2}M_{y2} & M_{x3}M_{y3}\\ 1 & 1 \end{array} \end{array}\right]$$

(2) $$\left[\begin{array}{c} \begin{array}{c} P{\text{'}}_{x}\\ P{\text{'}}_{y} \end{array}\\ \begin{array}{c} 0\\ 0 \end{array} \end{array}\right]=\left[\begin{array}{c} \begin{array}{cc} a & b\\ e & f \end{array}\;\begin{array}{cc} \;\;\;c & d\\ \;\;\;g & h \end{array}\\ \begin{array}{cc} 0 & 0\\ 0 & 0 \end{array}\;\begin{array}{cc} \;0 & 0\\ \;0 & 0 \end{array} \end{array}\right]\left[\begin{array}{c} \begin{array}{c} M{\text{'}}_{x}\\ M{\text{'}}_{y} \end{array}\\ \begin{array}{c} M{\text{'}}_{x}M{\text{'}}_{y}\\ 1 \end{array} \end{array}\right]$$

The positions (*M’*_x_, *M’*_y_) of the face and nose obtained from the web-camera image are applied to the (*P’*_x_, *P’*_y_) in the thermal image using the matrix of the geometric transform as indicated in Equation (2). The result is presented in Figure 6.

The adaptive boosting (Adaboost) algorithm [28] is used for detecting the face region in the web-camera image as indicated in Figure 6a. The center of both nostrils within the predetermined area inside the detected face region is detected by image binarization in the web-camera image. The region of the face and nose in the thermal camera image is defined using Equation (2) based on the regions of the face and center of the nose in the web-camera image, as indicated in Figure 6b. Based on the region of the center of the nose, 30 × 30 pixel regions of both cheeks are analyzed to compare the variation of FT before and after watching 3D content as indicated in Figure 7. By using a non-wearable camera system for FT measurement as illustrated in Figure 2, users’ convenience can be enhanced even though watching a display for a long time.

### 2.5. Quality Measurements for Obtaining the Weights for Each Modality

To measure a more accurate level of eye fatigue, we perform a quality measurement for four modalities, EEG signals, eye BR, FT, and SE score, based on a fuzzy system. The measured quality values of each modality are used as the weight values for combining the variations of each modality before and after watching 3D displays. Detailed explanations are presented in Section 2.6.

For the quality measurement, two features (*F_1_* and *F_2_*) are extracted from the values of each modality as presented in Table 2. These two features are used as the inputs for the fuzzy system that produces the quality (weight) values of each modality. In Table 2, each modality indicates the variation of EEG, BR, FT and SE before and after watching the 3D display.

As explained in Section 2.2, the beta band (13–30 Hz) in the frequency domain of the EEG data is analyzed in our research to measure eye fatigue. Watching 3D content is known to influence the power of the EEG signals in the beta band [6,7]. Consequently, the amplitudes of the beta band of each electrode of the EEG signal are compared before and after watching the 3D display [10]. P7, F7, and P8 of Figure 3 are selected as the dominant EEG nodes that indicate the 1st, 2nd, and 3rd significant differences before and after watching the 3D display based on the *t-*test *p*-value [10]. We define the difference of amplitudes between P7 and F7 and P7 and P8 as the two features (*F_1_* and *F_2_*) of the EEG signal for quality measurement as indicated in Table 2. If the quality of the measured EEG signal is acceptable, the values from the three nodes, P7, F7 and P8, are consistently similar, and the consequence of *F_1_* and *F_2_* of the EEG signal are minimized. However, if the quality of the measured EEG signal is poor, which is frequently caused by EEG signal noise related to the movement of the head or a facial muscle, the differences among the values from the P7, F7 and P8 nodes are considerable, which makes the influence of the *F_1_* and *F_2_* of the EEG signal significant.

**Table 2:** Two input features for producing the quality (weight) values of each modality.

Modality	Feature	Explanation of Feature
EEG	*F_1_*	Difference of amplitude between P7 and F7
	*F_2_*	Difference of amplitude between P7 and P8
BR	*F_1_*	Sum of scores by sub-block-based template matching in left and right pupil areas
	*F_2_*	Difference of number of black pixels between left and right pupil areas
FT	*F_1_*	Difference between the temperatures of left and right cheek areas
	*F_2_*	Level of in-plane rotation of face
SE	*F_1_*	User preference for watching 3D content
	*F_2_*	Number of users watching 3D movies

The two features (*F_1_* and *F_2_*) of the eye BR for quality measurement are the score by sub-block based on template matching and the difference of black pixels between the left and right pupil areas as indicated in Table 2. As explained in Section 2.3, the pupil region is detected by sub-block-based template matching in our research. The higher the matching score, the more accurate the detected area of the pupil. The accuracy detection of the pupil area is indispensable for the accurate measurement of the eye BR. Therefore, the sum of the two matching scores of left and right pupil areas is used as *F_1_* of the eye BR. As explained in Section 2.3, using sub-block-based template matching, the rough position of the pupil region is located. Then, the accurate boundary and center of the pupil are detected by an image binarization and ellipse-fitting method as illustrated in Figure 5. Therefore, if the detected pupil area is correct, the number of the black pixels in the left and right pupil regions in the binarized image is similar. Hence, the difference of the number of black pixels between the left and right pupil areas is used as *F_2_* of the eye BR. The lower the *F_2_* of the eye BR, the more accurate the detected area of the pupil is regarded, which represents a better quality of the eye BR.

We use the difference between the temperature of the left and right cheek areas as *F_1_* of the FT. As explained in Section 2.4, based on the region of the center of the nose, a 30 × 30 pixel region of both cheeks was analyzed for comparing variation of the FT before and after watching 3D content as indicated in Figure 7. Typically, the temperatures of the left and right cheek areas are similar because our experiments are performed indoors. A significant difference between the temperatures of the left and right cheek areas indicates inaccurate detection of the regions of both cheeks, which represents a lower quality of FT. As the *F_2_* of the FT, the level of in-plane rotation of the face is used. The level of in-plane rotation is calculated based on the positions of both detected nostrils. If the level is large, the possibility of inaccurate detection of the regions of both cheeks increases, which represents a lower quality of FT.

For the *F_1_* and *F_2_* of the SE, the user’s preference of watching 3D content and the number of users watching 3D movies are used. In the case of a higher user’s preference and greater number of users watching 3D movies, we can assume that the user is more accustomed to 3D content and he (or she) can perform a more accurate and objective SE. That is because the beginner typically requires time to adapt himself to watching 3D content, even with well-made 3D content, and it is difficult for him to perform objective SE during the adaptation time.

### 2.6. Obtaining the Weight Value Using Quality Measurements based on a Fuzzy System

**Figure 8:**
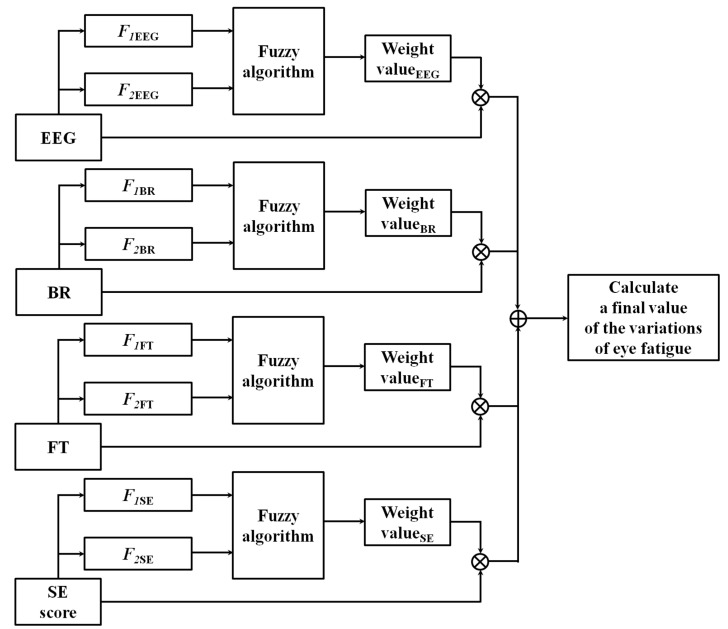
FBFM procedure for obtaining a final value of the variations of eye fatigue by weighted fusion of the values of four modalities.

With the features of each modality from Table 2, the optimal weight value of each modality is obtained using a fuzzy system as displayed in Figure 8. In Figure 8, the EEG, BR, FT, and SE scores represent the variations of EEG, BR, FT, and SE scores before and after watching a 3D display. Based on the optimal weight value, the final value of the variation of eye fatigue (before and after watching the 3D display) is calculated using a weighted fusion of the values of the four modalities as indicated in Figure 8.

**Figure 9:**
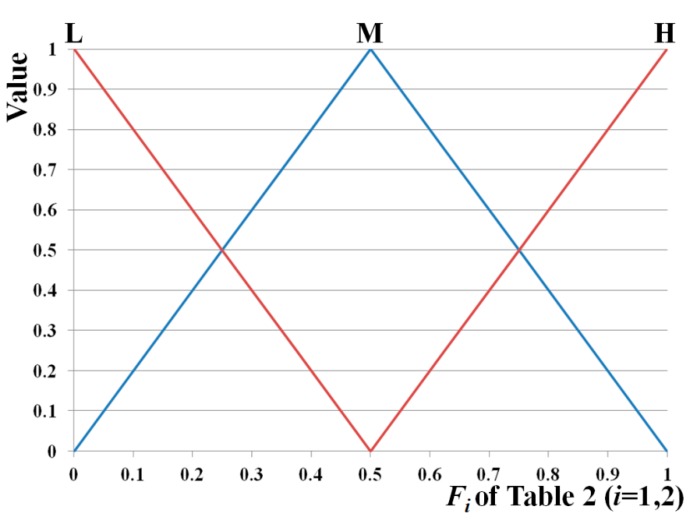
Input to fuzzy membership function.

**Figure 10:**
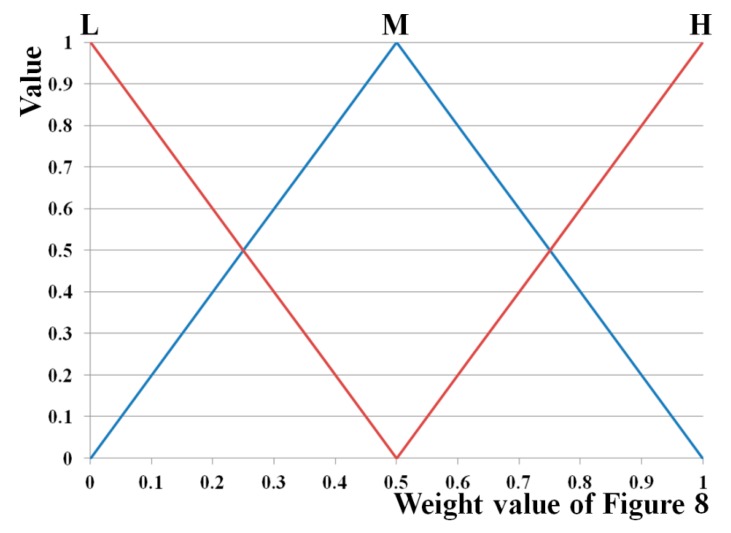
Output of fuzzy membership function.

To obtain the optimal weight value of each modality using the fuzzy system presented in Figure 8, the input and output membership functions of Figure 9 and Figure 10, respectively, are used. Input and output membership functions consist of three functions, low (L), middle (M), and high (H). In general, the membership function represents the distribution of the input or output values in a fuzzy system. We adopted the linear (triangular) membership function in our research because it has been widely used in fuzzy systems to consider the processing speed and complexity of problem to be solved [29,30,31].

Based on the characteristics of *F_1_* and *F_2_* of each modality explained in Section 2.5, we define the fuzzy rule table in Table 3. In Table 3, L, M, and H are low, medium, and high, respectively. The output of Table 3 represents the quality of each modality.

**Table 3:** Fuzzy rules tables for *F_1_* and *F_2_* of each modality, (**a**) EEG; (**b**) BR; (**c**) FT; (**d**) SE.

***F_1EEG_***	***F_2EEG_***	**Output**
L	L	H
	M	H
	H	M
M	L	H
	M	M
	H	L
H	L	M
	M	L
	H	L
**(a)**		
***F_1BR_***	***F_2BR_***	**Output**
L	L	M
	M	L
	H	L
M	L	H
	M	M
	H	L
H	L	H
	M	H
	H	M
**(b)**		
***F_1FT_***	***F_2FT_***	**Output**
L	L	H
	M	H
	H	M
M	L	H
	M	M
	H	L
H	L	M
	M	L
	H	L
**(c)**		
***F_1SE_***	***F_2SE_***	**Output**
L	L	L
	M	L
	H	M
M	L	L
	M	M
	H	H
H	L	M
	M	H
	H	H
**(d)**		

As explained in Section 2.5, if the quality of the measured EEG signal is high (H), the *F_1_* and *F_2_* of the EEG signal are low (L). If the quality of the measured EEG signal is low (L), the *F_1_* and *F_2_* of the EEG signal become large (H). The higher (H) the *F_1_* of the eye BR, the better the quality of the eye BR (H); the lower (L) the *F_2_* of the eye BR, the better the quality of the eye BR (H). If the quality of the measured FT is high (H), the *F_1_* and *F_2_* of the FT are low (L). If the quality of the measured FT is low (L), the *F_1_* and *F_2_* of the FT become large (H). For the *F_1_* and *F_2_* of the SE, a higher (H) for *F_1_* and *F_2_* represents a superior (H) SE quality.

**Figure 11:**
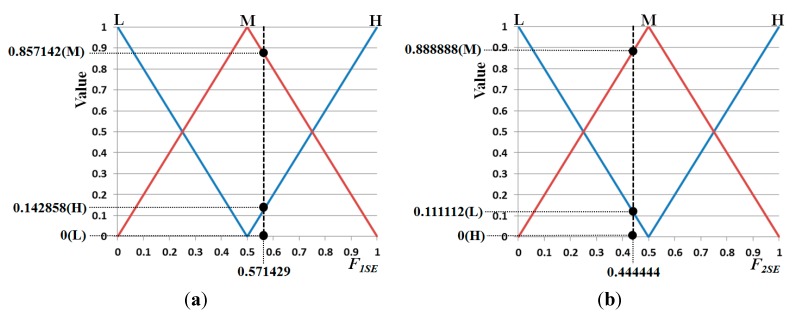
Example of obtaining the output value of the input membership function for SE, (**a**) *F_1_* of SE; (**b**) *F_2_* of SE.

In this section, we explain the procedure for obtaining the output value of the input membership function. For example, three outputs (0 (L), 0.857142 (M), 0.142858 (H)) are obtained based on the input membership function displayed in Figure 11a assuming that one input *F_1SE_* is 0.571429. Three outputs (0.111112 (L), 0.888888 (M), 0 (H)) are obtained indicated in Figure 11b assuming that one input *F_2SE_* is 0.444444. With these outputs, nine combination pairs of output using *F_1SE_* and *F_2SE_* are obtained as presented in Table 4.

**Table 4:** Example of nine combination pairs of output values of SE.

# of Combination Pair	Output of *F_1SE_*	Output of *F_2SE_*	Min Rule	Max Rule
1	0 (L)	0.111112 (L)	0 (L)	0.111112 (L)
2	0 (L)	0.888888 (M)	0 (L)	0.888888 (L)
3	0 (L)	0 (H)	0 (M)	0 (M)
4	0.857142 (M)	0.111112 (L)	0.111112 (L)	0.857142 (L)
5	0.857142 (M)	0.888888 (M)	0.857142 (M)	0.888888 (M)
6	0.857142 (M)	0 (H)	0 (H)	0.857142 (H)
7	0.142858 (H)	0.111112 (L)	0.111112 (M)	0.142858 (M)
8	0.142858 (H)	0.888888 (M)	0.142858 (H)	0.888888 (H)
9	0.142858 (H)	0 (H)	0 (H)	0.142858 (H)

As indicated in Table 4, based on the fuzzy rule table of Table 3d and min (or max rule), we can obtain nine values from the nine combination pairs. Min and max rules determine a minimum and maximum value between two outputs, respectively. For example with the first combination pair of (0 (L), 0.111112 (L)), 0 is selected by the min rule. Based on the fuzzy rule table of Table 3d (if L and L, then L), L is obtained. Therefore, 0 (L) is obtained by the min rule, which is called an inference value (IV) in our paper. In a similar fashion, nine IVs (0 (L), 0 (L), … 0 (H) of Table 4) are obtained from the nine combination pairs using the min rule. Further, nine IVs (0.111112 (L), 0.888888 (L), … 0.142858 (H) of Table 4) are obtained from the nine combination pairs using the max rule.

With these nine pairs of IVs, the optimal weight values for each modality of Figure 8 are calculated using a defuzzification method [32,33]. There are various defuzzification methods such as first of maxima (FOM), last of maxima (LOM), middle of maxima (MOM), mean of maxima (MeOM), and center of gravity (COG) [32,33].

**Figure 12:**
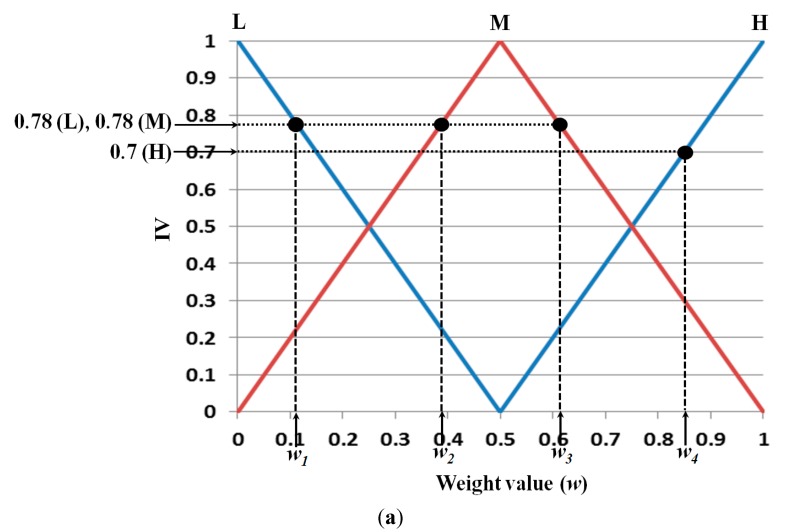

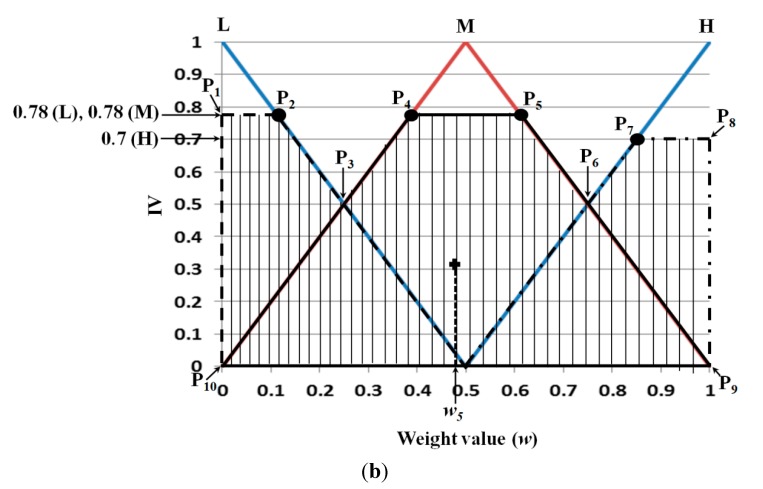
Examples of obtaining the weight values of Figure 8 by various defuzzification methods with nine IVs, (**a**) FOM, LOM, MOM, and MeOM; (**b**) COG.

We present examples of obtaining the weight values of Figure 8 by the various defuzzification methods with the IVs in Figure 12. In the following section, we explain the defuzzification methods with only three exemplary IVs for convenience. Assuming that the three IVs are 0.78 (L), 0.78 (M), and 0.7 (H), in Figure 12a, the minimum weight value (*w_1_*) among the weight values (*w_1_*, *w_2_*, and *w_3_*) calculated by the maximum IVs (0.78 (L) and 0.78 (M)) is selected as the optimal weight value using the FOM method. The LOM method selects the maximum weight value (*w_3_*) among the weight values (*w_1_*, *w_2_*, and *w_3_*) calculated by the maximum IVs (0.78 (L) and 0.78 (M)). The MOM method selects the middle value ((*w_1_*+*w_3_*)/2). The MeOM method selects the mean value ((*w_1_*+*w_2_*+*w_3_*)/3) as the optimal weight value. Finally, the COG method determines *w_5_* (the geometrical center of the polygonal region defined by ten points (P_1_, P_2_, … P_10_)) as the optimal weight value as indicated in Figure 12b.

### 2.7. Obtaining One Final Value of the Variations of Eye Fatigue Using Weighted Sum Method

Based on the optimal weight value (Weight value_i_, (i = EEG, BR, FT, SE) of Equation (3)) of each modality as indicated in Figure 12, we obtain the normalized weight value (NW_i_, (i = EEG, BR, FT, SE)) as indicated in Equation (3):

(3) $$NW_{\text{i}}=\frac{{\text{Weight value}}_{\text{i}}}{{\text{Weight value}}_{\text{EEG}}+{\text{Weight value}}_{\text{BR}}+{\text{Weight value}}_{\text{FT}}+{\text{Weight value}}_{\text{SE}}}$$

(i = EEG, BR, FT, SE)

(4) $$V_{eye\;fatigue}=NW_{\text{EEG}}\times V_{\text{EEG}}+NW_{\text{BR}}\times V_{\text{BR}}+NW_{\text{FT}}\times V_{\text{FT}}+NW_{\text{SE}}\times V_{\text{SE}}$$

Then, we obtain the final value of the variations of eye fatigue ($V_{eye\;fatigue}$) (before and after watching 3D display) using a weighted sum method based on the normalized weight values (*NW*_i_, (i = EEG, BR, FT, SE)) and the variations (*V*_i_, (i = EEG, BR, FT, SE)) of the input value of each modality (before and after watching 3D display) using Equation (4).

## 3. Experimental Setup and Results

To simultaneously acquire the data for EEG, FT, and eye image for BR without time delay, two desktop computers with an additional laptop computer were used. The first desktop computer (used to capture the images of both eyes using a high-speed camera) had a 3.07 GHz CPU (Intel (R) Core (TM) i7 CPU 950) and 6 GB RAM. The second desktop computer (used to acquire the EEG signals using the Emotiv EPOC headset) had a 2.33 GHz CPU (Intel (R) Core (TM) 2 Quad CPU Q8200) and 4 GB RAM. The laptop computer (used to capture the images from the web-camera and thermal camera) had a 2.50 GHz CPU (Intel (R) Core (TM) i5-2520M) and 4 GB RAM. The proposed method for measuring eye fatigue was implemented with a C++ program using the Microsoft Foundation Class (MFC) and OpenCV library (ver. 2.3.1).

A detailed description of the participants and sample images for the experiments can be found in [10]. A group of 15 subjects (male: 12, female: 3) participated in the experiments (average age: 26.89, standard deviation: 1.96). We obtained written and informed agreements from each participant. We used 3D content entitled “*Summer in Heidelberg*” for our experiments. It consists primarily of landscape scenes as illustrated in Figure 2. We obtained the permission of the copyright owner of the content [34]. The highest brightness of the display used for experiments was 99.546 cd/m^2^ and the luminance of the room was measured as 321 lux.

**Figure 13:**
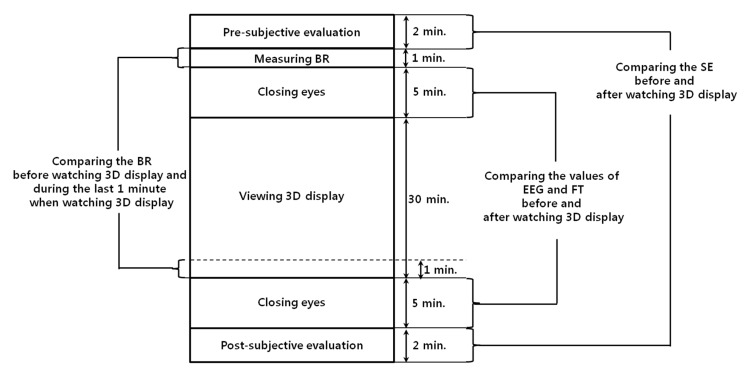
Experimental procedures.

Figure 13 presents the experimental procedures. To guarantee the accuracy of the eye fatigue measurement, the variations of EEG signal, eye BR, and FT were measured with SE before and after watching the 3D display [10]. To measure the natural BR in the last minute of Figure 13, we did not provide any artificial indication or instruction for the user’s alertness. There were no dozing or drowsy participants in the experiments. To compare the status of each participant before and after watching the 3D display, an SE was also performed with the questionnaire form displayed in Table 5 using a 10-point scale (1: Not at all~10: Yes, very much). Based on previous research [10,35], these questions were made to measure the status of each participant.

**Table 5:** Questionnaire for SE.

Six questions for SE
I have difficulties seeing
I have a strange feeling around my eyes
My eyes feel tired
I feel numb
I feel dizzy looking at the screen
I have a headache

In previous research [10], the variations of EEG data, eye BR, FT, and an SE score caused by eye fatigue before and after watching 3D TV were measured. However, the researchers did not obtain one final value for the variation of eye fatigue by combining the values of the EEG data, eye BR, FT, and an SE score considering the qualities of each modality. Unlike [10], we propose a method of obtaining one final value for the variation of eye fatigue based on FBFM considering the qualities of EEG data, eye BR, FT, and an SE score.

Based on FBFM, the sum of the correlation values with other data was calculated as presented in Table 6. The sum of the correlation values with other data was highest when obtaining the final value of the variation of eye fatigue using the MAX rule and COG defuzzification method. Therefore, all the following experiments using FBFM were performed based on the MAX rule and COG defuzzification method.

**Table 6:** Sum of the correlation values with other data for EEG, BR, FT, and SE according to various defuzzification methods and min or max rule.

Method		EEG	BR	FT	SE	Sum of the Correlation Values
Min rule	FOM	0.3969	0.2742	0.5977	0.663	1.9318
	MOM	0.3405	0.3312	0.6202	0.7204	2.0123
	LOM	0.2908	0.3733	0.6308	0.7581	2.053
	MeOM	0.3405	0.3312	0.6202	0.7204	2.0123
	COG	0.298	0.4255	0.6736	0.7683	2.1654
Max rule	FOM	−0.0275	0.4176	0.6563	0.6871	1.7335
	MOM	0.0914	0.5136	0.7091	0.8094	2.1235
	LOM	0.1508	0.5564	0.681	0.8486	2.2368
	MeOM	0.1315	0.5254	0.7002	0.8303	2.1874
	COG	0.2268	0.5464	0.6381	0.8413	**2.2526**

For the next experiment, we measured the gradient, R^2^, and correlation value between the value of the eye fatigue using the proposed FBFM and each of the EEG, BR, FT, and SE data as presented in Table 7. Linear regression is the usual approach for obtaining the line that is optimally fitted with the data distribution in 2D space. The gradient and R^2^, which are calculated from the line, represent the measure of steepness of data distribution and the confidence level of the fitted data in the predicted regression line, respectively. In general, the higher the data reliably fits the regression line, the larger the R^2^ value becomes [10]. The range of the correlation value is represented from −1 to 1 [36]. −1 and 1 represent strong negative and strong positive relationships, respectively. If the correlation value is 0, the data is completely uncorrelated.

**Figure 14:**
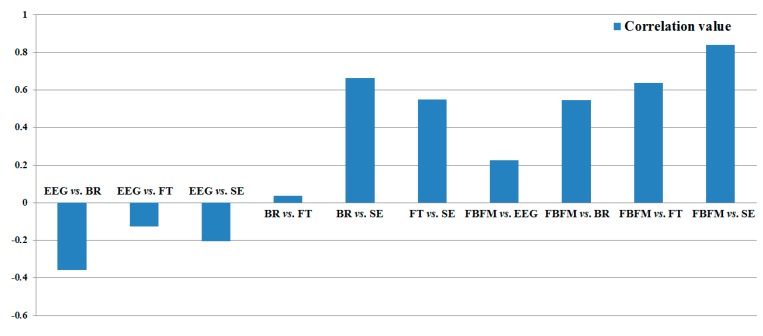
Result of correlation value between each modality and the value of eye fatigue using the proposed FBFM.

In Table 7, we also include the gradient, R^2^, and correlation value of each modality, which were measured in [10]. As illustrated in Table 7 and Figure 14, the correlation and R^2^ values between the value of eye fatigue using the proposed FBFM and SE are the highest and between the BR and FT are the lowest.

**Table 7:** Results of gradient, R^2^, and correlation value between each modality and the value of eye fatigue using the proposed FBFM. Value of eye fatigue using the proposed FBFM is labeled FBFM.

Method		Gradient	R^2^	Correlation Value
Previous research [10]	EEG *vs.* BR	−0.33	0.1285	−0.3585
	EEG *vs.* FT	−0.1154	0.0156	−0.125
	EEG *vs.* SE	−0.1584	0.0421	−0.2052
	BR *vs.* FT	0.0381	0.0014	0.038
	BR *vs.* SE	0.5582	0.4427	0.6653
	FT *vs.* SE	0.4593	0.3015	0.5491
Proposed method	FBFM *vs.* EEG	0.4804	0.0515	0.2268
	FBFM *vs.* BR	1.0654	0.2986	0.5464
	FBFM *vs.* FT	1.2478	0.4072	0.6381
	FBFM *vs.* SE	1.3762	0.7078	0.8413

To compare the correlation values between each data and the value using FBFM, we obtained the correlation matrix presented in Table 8. The relationships between the EEG and other data (BR, FT, and SE) were negatively analyzed because EEG signals contain noise such as the movement of the head or facial muscles. As indicated in Table 8, the absolute correlation value between FBFM and SE is highest (0.8413), and that between BR and FT is lowest (0.038) for the measurement of eye fatigue for the 3D content.

As illustrated in Table 7 and Figure 14, the gradient, R^2^, and correlation values obtained using the proposed FBFM and SE are greater than those of the previous method (EEG *vs.* SE, BR *vs.* SE, and FT *vs.* SE) [10]. The values using the proposed FBFM and FT are greater than those using the previous method (EEG *vs.* FT, BR *vs.* FT, and SE *vs.* FT). Similarly, the majority of the gradient, R^2^, and correlation values obtained using the proposed FBFM and BR and the proposed FBFM and EEG are also greater than those using the previous method. From this, we can confirm that the eye fatigue value obtained using the proposed FBFM is more correlated with each modality, EEG, BR, FT, and SE, compared to that using the previous method using each modality without fusion [10]. This means that the credibility of the eye fatigue value using the proposed FBFM is higher than that of using each modality without fusion and that a more accurate eye fatigue can be measured using the proposed FBFM compared to the previous method.

**Table 8:** Correlation matrix of four measured data and the value using FBFM before and after (or in the final one minute) watching 3D content.

	EEG	BR	FT	SE	FBFM	Sum of the Correlation Values with Other Data
EEG	1	−0.3585	−0.125	−0.2052	0.2268	−0.4619
BR	−0.3585	1	0.038	0.6653	0.5464	0.8912
FT	−0.125	0.038	1	0.5491	0.6381	1.1002
SE	−0.2052	0.6653	0.5491	1	0.8413	1.8505
FBFM	0.2268	0.5464	0.6381	0.8413	1	2.2526

**Figure 15:**
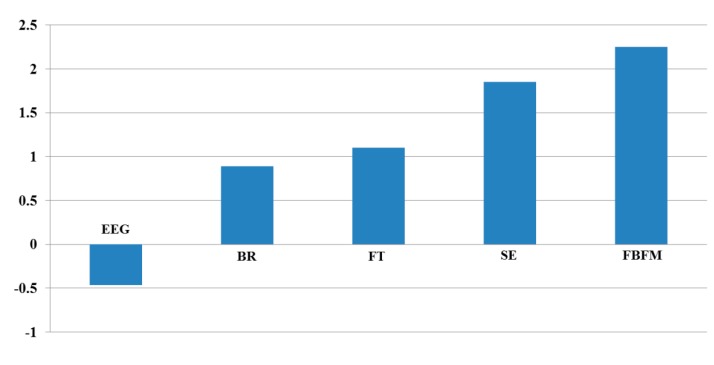
Result of the sum of the correlation values with other data.

To quantitatively assess the correlation values between each data, we added the correlation values excluding the auto-correlation value of 1 (for example, correlation value between EEG and EEG). As presented in Table 8 and Figure 15, the correlation of the proposed method (FBFM) with other data is highest (2.2526). Those of the SE, FT, BR, and EEG with other data are second, third, fourth, and fifth highest, respectively. From these results, we can confirm that the value of eye fatigue using the proposed FBFM is more correlated with the value of each modality compared to not combining each modality. This indicates that the value of eye fatigue using the proposed FBFM is more credible than not combining each modality.

In the next analysis, we performed an independent two-sample t-test, which has been widely used as a statistical hypothesis test [37], with the variations of eye fatigue using the proposed FBFM. The null-hypothesis (the two scores of eye fatigue using the FBFM are equal before and after watching the 3D display) was used for the t-test. The thresholds for the confidence level of 99% and 95% used were 0.01 and 0.05, respectively. In general, if the calculated p-value is less than the threshold of 0.01 or 0.05, the null-hypothesis is rejected based on the confidence level of 99% or 95%, respectively [37]. This mean that the two scores of eye fatigue using the FBFM before and after watching the 3D display are significantly different based on the confidence level. Experimental results determined that the calculated p-value of the FBFM is 0.0471, which is less than the threshold of 0.05. Therefore, we can confirm that with a confidence level of 95%, the two eye fatigue scores using FBFM were significantly different before and after watching the 3D display.

For the last test, we analyzed the variations of the eye fatigue using the FBFM before and after watching the 3D display using the effect size in descriptive statistics. The effect size is usually accepted as a descriptive statistic and has been widely used to represent the power of a measured phenomenon in statistics [38]. Based on previous research [39], we defined Cohen’s *d* values of 0.2, 0.5, and 0.8 as small, medium, and large, respectively. Cohen’s *d* value is calculated based on the difference between two means divided by the standard deviation of the measured data. If the calculated Cohen’s *d* value is close to 0.2, the measured data is regarded as having small effect size. If the value is close to 0.8, the measured data can be regarded as having large effect size. Experimental results determined that Cohen’s *d* value was 0.7704, which is closer to 0.8, compared to 0.2 or 0.5, and we can confirm that the variations of the eye fatigue using FBFM before and after watching the 3D display can be regarded as having a large effect size.

**Figure 16:**
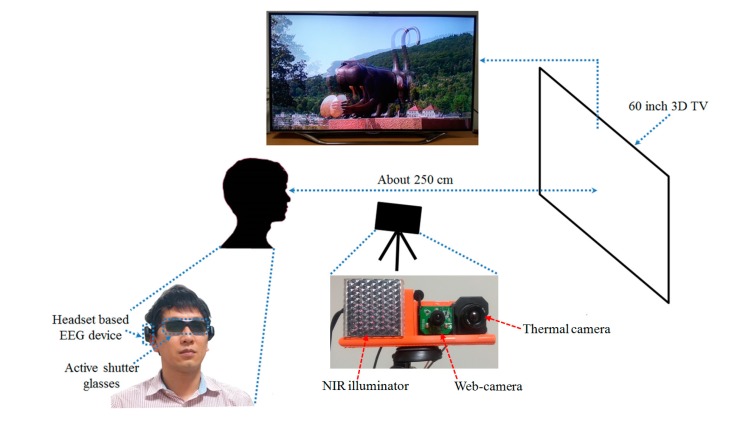
Simplified capturing system for measuring eye fatigue on 3D display.

**Figure 17:**
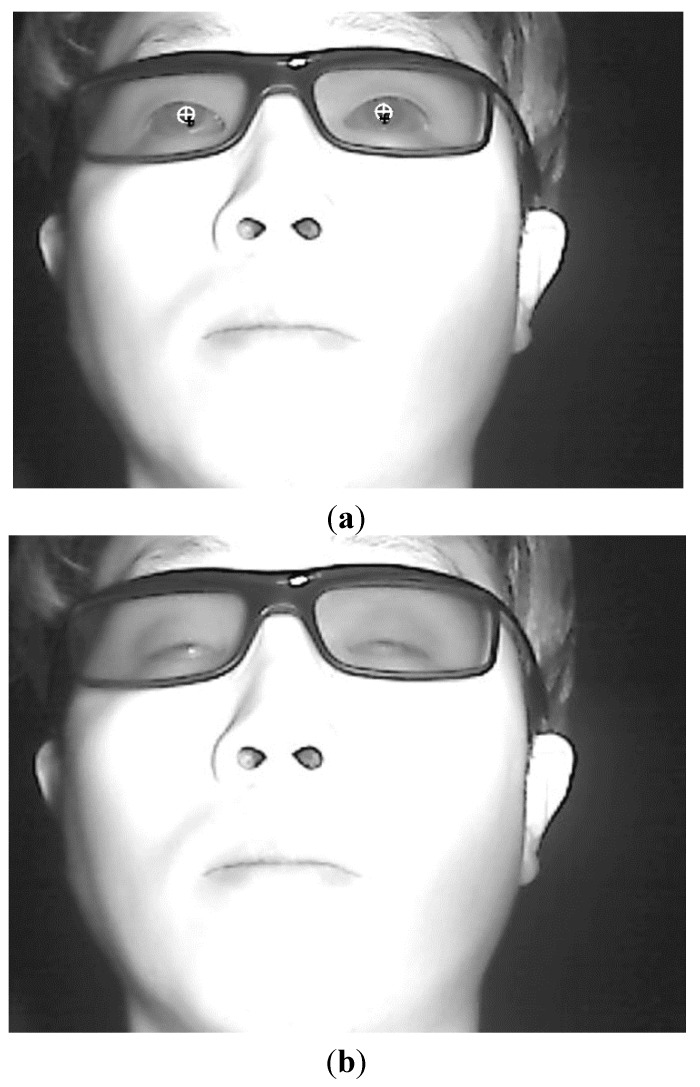

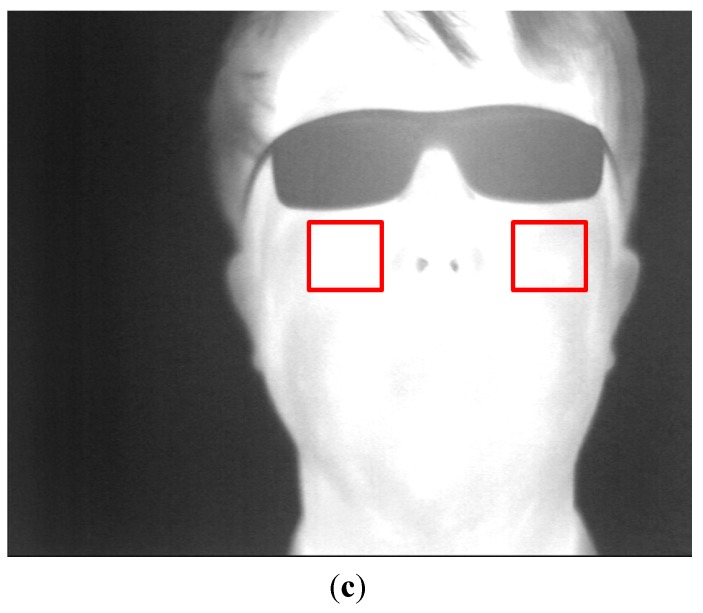
Examples of captured image by the simplified capturing system, (**a**) Detected pupil center and specular reflection center in case of open eye in the image by web-camera; (**b**) Closed eye in the image by web-camera; (**c**) Image by the thermal camera for defining the cheek regions for measuring facial temperate.

In addition, we simplified the capturing system of Figure 2 as follows. In the simplified system, we combined two functionalities of measuring eye BR by the high-speed camera and detecting face and nostril for defining the cheek regions (where facial temperature is measured) by the web-camera of Figure 2. That is, we removed the high-speed camera of large size, and made these two functionalities be performed by one web-camera. The revised capturing system is shown in Figure 16, and the captured images by this system are shown in Figure 17. Like the previous system of Figure 2, in order to detect accurate pupil area through active shutter glasses, NIR illuminator is also used in the revised system of Figure 16. By attaching the NIR illuminator to the left side of the web-camera as shown in Figure 16, we made the small capturing system whose size is 18 × 7 × 5 cm^3^ (width × height × depth) and whose weight is about 330 g.

In order to prove that the performance of this simplified capturing system of Figure 16 is similar to that of previous system of Figure 2, we measured the accuracies of eye BR by this system. Because the eye BR is measured by counting the number of closed eyes for a time duration of one minute, the accuracy of eye BR can be measured by Type 1 and 2 errors. Type 1 error represents the error of misclassifying open eye into close one whereas Type 2 error shows the error of misclassifying close eye into open one. Experimental results (by this simplified capturing system of Figure 16 with 15 people) showed that the Type 1 and 2 errors are 99.2% and 99.1%, respectively, which are similar to those (99.2% and 99%) by the previous system of Figure 2.

As the next test for proving that the performance of this simplified capturing system is similar to that of previous system of Figure 2, we measured the accuracies of detected face and nostril in web-camera image and detected cheek regions for measuring facial temperature in thermal image. Experimental results (by this simplified capturing system of Figure 16 with 15 people) showed that the accuracies of detected face, nostril, and cheek regions are 99.9%, 99.8% and 99.5%, respectively, which are similar to those (99.9%, 99.7% and 99.3%) by the previous system of Figure 2. Because EEG signal is measured by the previous EEG measurement device of Figure 2, the performance of EEG measurement by the system of Figure 16 is same to that of previous system of Figure 2.

**Figure 18:**
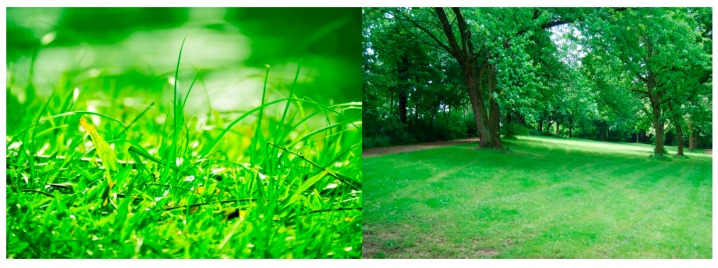
Examples of two images which were used for measuring the bias value of the change of BR caused by being exposed to the NIR illuminator.

In our research, we normalized the BR considering the bias in our original experimental results. That is, in order to measure the bias value which represents the change of BR caused by being exposed to the NIR illuminator (excluding the change effect of BR caused by eye fatigue of 3D display), we performed the experiment with two images which are comfortable to user’s eyes. Based on previous research that the green color can be more comfortable to user’s eye [40], we used two sample images for experiment. These images do not have any copyright [41], and they are shown in Figure 18.

In detail, before performing the experiments of Figure 13, we measured the BR of 15 participants (who took part in our experiments of Figure 13) with the two images of Figure 18 under same experimental environment (luminance of the room, display size, and distance of user to display, *etc.*) to that of Figure 13.

At first, BR was measured for 1 min. before each people looked at the two images, which corresponds to measuring BR before viewing 3D display of Figure 13. Then, each people closed eyes for 5 min. like the experimental procedure of Figure 13. After that, BR was measured again in the last 1 min. while each people looked at the images for 30 min., which corresponds to measuring BR of the last 1 min. of viewing 3D display of Figure 13. After that, each participant took sufficient rest before doing the experiments of Figure 13.

**Figure 19:**
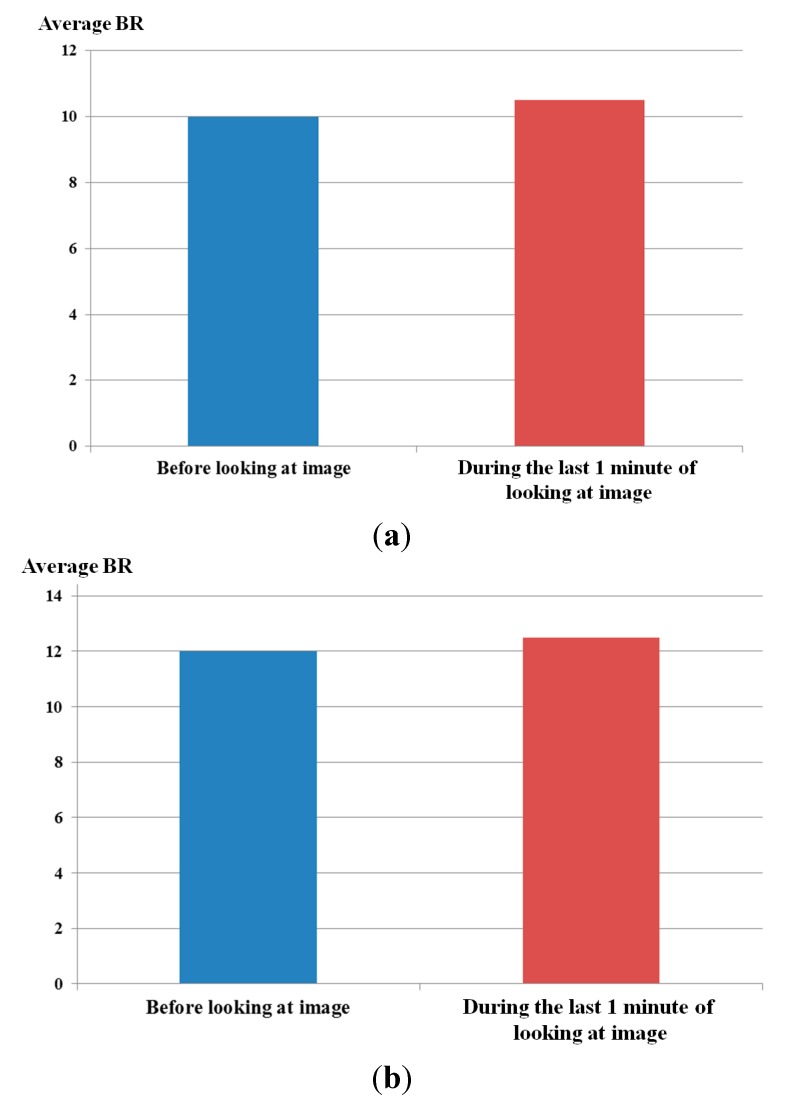
Two average graphs of BR before looking at the images and in the last 1 min. while looking at the images. Graphs when people looked at (**a**) the left image of Figure 18, (**b**) the right image of Figure 18.

From this experiment with two images of Figure 18, we obtained the two average graphs of BR of 15 participants as shown in Figure 19. In both cases of Figure 19a,b, the BRs during the last 1 min of looking at the image are a little increased compared to those before looking at the image. Then, we obtained the average graphs of Figure 19a,b, and two average BRs (before and during the last 1 min of looking at the image, respectively) were obtained. The difference between these two average BRs was used as the bias value which represents the change of BR caused by being exposed to the NIR illuminator (excluding the change effect of eye BR caused by eye fatigue of 3D display).

By conclusion, we already reflected this bias value to the measured eye BRs of our original experiments of Figure 13, from which we obtained the correct BRs which were not biased to the change of BR caused by being exposed to the NIR illuminator.

## 4. Conclusions

In this study, we proposed a new FBFM for the assessment of eye fatigue caused by viewing 3D content based on multimodal measurements. To measure a more accurate eye fatigue, based on the quality measurements of EEG signals, BR, FT and SE score, we obtained the optimal weight values of each modality using a fuzzy system. The final weighted sum of each modality was calculated to measure a more accurate level of eye fatigue. Experimental results confirm that the value of eye fatigue using the proposed FBFM is correlated higher with the value of each modality compared to cases when not combining each modality. This indicates that the value of eye fatigue using the proposed FBFM is more credible than that of not combining each modality. Moreover, the credibility of the variations of eye fatigue using FBFM before and after watching the 3D display was verified based on a t-test and descriptive statistics analysis using the effect size. In future work, we will combine additional modalities such as EOG and electromyography (EMG) to measure a more accurate value of eye fatigue. Further, we will adapt the proposed method of eye fatigue measurement to various smart phone and tablet computer displays.
